# Statistical Computations Underlying the Dynamics of Memory Updating

**DOI:** 10.1371/journal.pcbi.1003939

**Published:** 2014-11-06

**Authors:** Samuel J. Gershman, Angela Radulescu, Kenneth A. Norman, Yael Niv

**Affiliations:** 1 Department of Brain and Cognitive Sciences, Massachusetts Institute of Technology, Cambridge, Massachussetts, United States of America; 2 Department of Psychology and Princeton Neuroscience Institute, Princeton University, Princeton, New Jersey, United States of America; Indiana University, United States of America

## Abstract

Psychophysical and neurophysiological studies have suggested that memory is not simply a carbon copy of our experience: Memories are modified or new memories are formed depending on the dynamic structure of our experience, and specifically, on how gradually or abruptly the world changes. We present a statistical theory of memory formation in a dynamic environment, based on a nonparametric generalization of the switching Kalman filter. We show that this theory can qualitatively account for several psychophysical and neural phenomena, and present results of a new visual memory experiment aimed at testing the theory directly. Our experimental findings suggest that humans can use temporal discontinuities in the structure of the environment to determine when to form new memory traces. The statistical perspective we offer provides a coherent account of the conditions under which new experience is integrated into an old memory versus forming a new memory, and shows that memory formation depends on inferences about the underlying structure of our experience.

## Author Summary

When do we modify old memories, and when do we create new ones? We suggest that this question can be answered statistically: The parsing of experience into distinct memory traces corresponds to inferences about the underlying structure of the environment. When sensory data change gradually over time, the brain infers that the environment has slowly been evolving, and the current representation of the environment (an existing memory trace) is updated. In contrast, abrupt changes indicate transitions between different structures, leading to the formation of new memories. While these ideas fall naturally out of statistical models of learning, they have not yet been directly tested in the domain of human memory. In this paper, we describe a model of statistical inference that instantiates these ideas, and test the model by asking human participants to reconstruct previously seen visual objects that have since changed gradually or abruptly. The results of this experiment support our theory of how the statistical structure of sensory experiences shapes memory formation.

## Introduction

How does the brain take a continuous stream of sensory inputs and translate it into stored memories? Theorists have offered radically different answers to this question. According to biologically inspired theories (e.g., [1]–[3]), input patterns are continuously assimilated into a distributed network of interconnected neurons via modification of synaptic connections. When a network trained in this fashion is allowed to run freely or with partial input, it will converge to one or more stable configurations–*attractors*–corresponding to blends of stored input patterns. This view of memory asserts that experiences are not stored individually, but rather overlaid on one another. Many modern psychological theories of memory (e.g., [4]–[6]) adopt a diametrically opposed view: Input patterns are stored separately, and memory blending, if it occurs, happens at retrieval rather than during storage (though see [7]–[9] for notable exceptions which allow memory traces to be modified by multiple input patterns).

One way to approach this question is to consider the information processing problem being solved by the memory system. If we were to design a brain, how would it parse experience into memory traces? This exercise in “rational analysis” [10] leads us to a statistical formulation of the memory storage problem. We propose that the memory system is designed to facilitate optimal predictions under a particular generative model of the environment. According to this generative model (see also [11], [12]), the environment tends to change slowly over time, but occasionally jumps between completely different “modes.” For instance, while the temperature can fluctuate slowly within different parts of a building, going outside is characterized by very different (but also slowly changing) temperatures than those that were in effect indoors. Stored memories then correspond to inferences about the latent modes (e.g., we can recall the general temperature inside the building, and separately, the outdoor temperature), and input patterns are clustered together if they are inferred to have been generated by the same mode. This theory retains the idea from the cognitive psychology literature that the memory system contains multiple traces, but assumes that each trace may be a blend of several input patterns, as is the case for many neural network models.

Memories are no doubt stored at many resolutions: while you might have a general memory of being cold when outside and warm when inside, you will also probably remember precisely whether you wore a hat to combat the cold. Following traditional psychological models, we claim that separate traces for each input pattern are stored at the finest-grained, most “episodic” resolution. Layered on top of these episodic separate traces are more general traces that serve to organize memory retrieval and form predictions of the future. At this coarser resolution, experience must be parsed into separate traces or combined into more general traces. The goal of our theory is to illuminate the laws governing memory parsing. Depending on the statistical structure of the environment, this parsing process will produce traces that appear more or less “semantic,” in the sense that they aggregate information over individual episodes [13], [14]. In order to avoid cumbersome terminology, we will henceforth use “traces” to refer to those traces formed as the result of parsing at the coarser-grained resolution of memory.

We tested our theory using a novel behavioral task that allows us to assess qualitatively whether participants store different stimuli in one or several memory traces. We presented dynamically changing visual stimuli to participants, and subsequently asked them to reconstruct one of the previously presented stimuli from memory. When the stimuli changed gradually, the reconstructions suggested that participants had, to some extent, inferred a single dynamical mode and thus formed one memory trace in which different instances interfered with each other. In contrast, when the stimuli changed abruptly, participants' behavior suggested that they had inferred two dynamical modes, one before the abrupt change and one after. This resulted in less interference between stimuli experienced before and after the change, and reconstruction of stimuli presented before the change was more accurate.

### Background

Recent psychophysical studies have explored the dynamics of memory updating by presenting participants with sequences of stimuli and then probing their ability to discriminate between different stimuli in the sequence. The logic of these studies is that if the stimuli are assimilated into the same dynamical mode, then they will be perceived as being more similar, compared to a situation where they are segmented into different modes. For example, Wallis and Bülthoff [15] presented participants with a rotating face that gradually morphed into a different face. Compared to a condition in which the morphs were presented in a mixed (scrambled) order, participants in the gradual morph condition were more prone to perceive the final face as belonging to the same person as the original face. Similar findings were reported by Preminger and colleagues [16], [17] using a variety of memory tests.

These psychophysical observations are complemented by neurophysiological studies of spatial representation in the rodent hippocampus. Many neurons in the CA3 subfield of the hippocampus respond selectively when the animal is in a particular region of space, and are therefore known as “place cells” [18]. We can apply the same logic used in the aforementioned psychophysical studies to the hippocampal representation of space [19], asking whether morphing one environment into another will lead to gradual changes in place cell firing rate (indicating a gradually changing spatial memory) or a global remapping of place fields (indicating the formation of a new memory). Leutgeb et al. [20] and Wills et al. [21] had rats explore a set of enclosures whose shape varied from a square to a circle (including intermediate shapes). Gradually changing the enclosure shape (the “gradual” protocol) resulted in gradual changes in place fields [20], whereas presenting the same series of enclosures in a scrambled order (the “mixed” protocol) resulted in global remapping – enclosures that were more similar to the circle than to the square tended to elicit one set of place fields, and enclosures that were more similar to the square than to the circle tended to elicit a distinct set of place fields [21]. As with the psychophysical findings described above, these results highlight the importance of sequential structure in guiding memory organization; the same stimuli can elicit very different internal representations depending on the order in which they are presented.

Using a Hopfield network to encode the input patterns, Blumenfeld et al. [22] proposed a “salience-weighted” modification of the standard Hebbian learning rule to model these findings. Intuitively, the salience weight encodes a prediction error or novelty signal that indicates the extent to which none of the network's existing attractors match the current input pattern. Formally, the salience weight is the Hamming distance between the input pattern and the network state after one step of dynamics; the salience weight is updated incrementally after each input pattern so as to smooth across recent history. A large salience weight promotes the formation of a new attractor based on the current input. For our purposes, the key idea to take away from this model is that prediction errors are useful signals for determining when to infer new memory modes (see also [23]–[26]). In the network explored by Blumenfeld et al., a new attractor is only formed if the prediction error is sufficiently large, but how large is “sufficient”? In the next section, we place these ideas within a statistical framework, which allows us to specify the prediction error threshold in terms of probabilistic hypotheses about the environment.

### The statistical framework

The essence of our approach is captured by the following generic assumption about the environment: Properties of the environment usually change gradually, but occasionally undergo “jumps” that reflect a new underlying state of affairs [11], [12]. Returning to the temperature example, when you walk around outside, you may experience gradual changes in temperature over the course of the day. If you step into a building, the temperature may change abruptly. In predicting what the temperature will be like in 5 minutes, you might then generalize from one outdoor location to another, but not between the indoor location and outdoor locations. Thus, our generalizations depend strongly on how we segment our observations; cognitively speaking, one can view each segment as a memory trace that aggregates those observations assigned to the segment. The empirical data reviewed in the previous section are consistent with the idea that the brain is attuned to abrupt changes in the state of the environment.

The problem of estimating the current state of a hidden variable given previous sensory measurements is known in engineering as *filtering*. The classic example of a filtering algorithm is the Kalman filter (KF; [27]), which is the Bayes-optimal estimator under the assumption that the environment evolves according to a linear-Gaussian dynamical system (LDS) –i.e., the state of the environment changes gradually and noisily over time. By design, this model cannot account for large sporadic jumps and periods of gradual change between them.

One way to model jumps is to posit a collection of different “dynamical modes”, each corresponding to a slowly changing LDS, and allow the generative process to switch between them stochastically. This is known as a switching LDS, and its corresponding Bayes-optimal estimator is the switching KF. However, for real-world sensory measurements, it is not reasonable to specify the number of possible modes in advance. We therefore adopt a Bayesian infinite-capacity (nonparametric) generalization of the switching LDS based on the Dirichlet process [28], which allows the number of modes to expand as necessary as measurements are collected (another dynamical model that could capture jumps within a single mode is a random walk with a heavy-tailed distribution on step size, such as a Lévy flight [29]).

The infinite-capacity prior over modes leads to an intuitive interpretation in terms of memory traces: Each mode clusters together a number of individual observations, and thus can be identified with a temporally extended episodic memory trace such as the memory of the temperature outside. The number of such modes is essentially unlimited. However, because in our model small numbers of modes have higher probability *a priori*, the result is that the memory system tries to account for its observations as parsimoniously as possible by using existing modes to explain multiple observations. This leads to potential modification of existing modes each time a new observation is assigned to them, and sporadic creation of new modes. Below we describe this model formally.

## Results

We first propose a normative computational model that can account for the psychophysical and neural findings discussed in the Introduction. We then describe a new psychophysical experiment that tests the predictions of our model.

### Generative model

Let 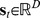 denote a set of sensory measurements at time *t*, arising from unobservable state variables 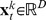, where *k* indexes modes. For instance, the observation may be the current temperature, and the state variables are the air pressure, cloud coverage, inside/outside location, air conditioner, thermostat status, and many other direct causes of temperature. Let 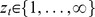 denote the mode active at time *t*. This mode specifies particular state-space dynamics, for instance, a mode corresponding to being indoors with the air conditioning on (which specifies the dependence of temperature on thermostat settings), another corresponding to air conditioning being off, another to being outside in the shade, etc.

Our model assumes that measurements (observations) are generated according to the following stochastic process. For each time point *t*:

1. Draw a mode 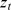 from a *sticky Chinese restaurant process* prior [30]:
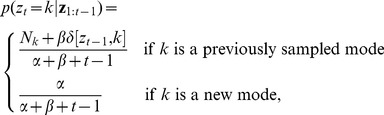
(1)where 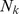 is the number of previous timepoints in which mode *k* was drawn, 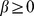 is a stickiness parameter that governs mode persistence, and 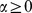 is a concentration parameter that specifies the probability of drawing a completely new mode. When 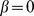, Eq. 1 generates a partition of trials to modes 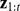 that corresponds to the distribution over partitions induced by a Dirichlet process [31]. This prior assigns higher probability to partitions with a small number of dynamical modes, and hence expresses a “simplicity principle” [32] –all else equal, sensory data are more likely to be generated by a simpler environment, comprised of fewer modes. When 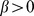, modes tend to persist over multiple consecutive time points, with *β* controlling the strength of this persistence.
2. If 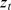 is a new mode, draw the state variable 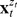 from a Gaussian base measure: 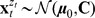, where 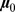 is the prior mean and 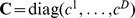 the covariance matrix of the state variables.
3. Diffuse the state variables for each active mode: 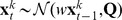, where 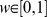 is a decay term and 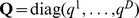 is the diffusion noise covariance matrix. The diagonal terms of Q determine the rate of change: larger values of 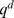 induce more rapid change along dimension *d*. Note that the state variable for a mode (once it is activated for the first time) evolves even when that mode is no longer active.
4. Emit sensory measurements 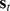 from a Gaussian centered on the state of the currently active mode 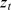: 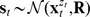, where 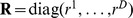 is the sensory noise covariance matrix.

This generative model is a simplification of the nonparametric switching LDS described in [28].

To summarize the generative model: The hidden state diffuses gradually until a jump occurs; this jump can be either to a previously activated mode, or to a new mode (in which case a new starting point is drawn for that mode, from a Gaussian prior). The concentration parameter 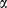 controls the probability that a new mode will be activated: Larger values of 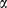 result in more modes, and if 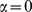, there are no jumps and we obtain a special case of the standard LDS formulation. The stickiness parameter *β* encourages modes to persist over time; when 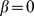, we recover the original Chinese restaurant process [33]. The diffusion variances 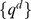 control the rate of change within a mode: Larger values of 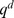 result in faster change. The sensory noise variance 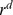 controls the informativeness of the observations about the hidden state: As 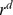 increases, the sensory measurements become noisier and hence convey less information about the hidden state.

### Bayesian inference with an infinite-capacity switching LDS

Given the generative model, the filtering problem is to infer the posterior distribution over the state variable 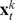 for each mode 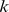 given the history of sensory measurements 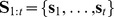. This computation is given by:
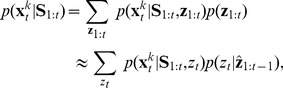
(2)


where 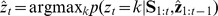. This corresponds to a “local” approximation [34]–[36] that maintains only a single high probability partition 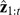 of previous observations to hidden causes. This partition is then used to calculate the probability of the current trial being drawn from each of the latent causes 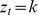 by combining the sticky Chinese restaurant process prior (Eq. 1) and the likelihood (conditional on the partition and the previous observations) of the current state vector 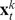. Although we could have used more sophisticated methods (e.g., particle filtering) to approximate the marginalization, this method works well on the examples we consider, and is much faster, making it easier to fit to behavioral data.

We now describe how to compute each of the components in Eq. 2. The conditional distribution 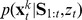 is a Gaussian, with mean 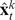 and covariance 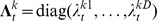, updated according to:
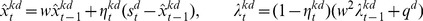
(3)


for each dimension *d*, where the estimated mean and variance for a new mode *k* are 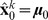 and 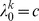 (respectively) and the step size (or learning rate) *η*, also known as the *Kalman gain*, is
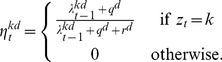
(4)


Using the local approximation described above, the posterior over mode assignments is given by:
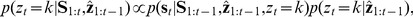
(5)


where the second term is the prior (Eq. 1), and the first term is the likelihood:
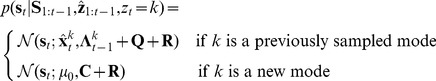
(6)


where “new mode” refers to the first mode that has never been active before time *i*. This completes the description of our inference algorithm, which we refer to as the *Dirichlet process Kalman filter* (DP-KF).

Viewed as a mechanistic psychological model, the DP-KF assumes that the memory system keeps track of two kinds of traces: episodic traces encoding the sensory stimulus at each time point (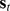), and more general traces that encode summary statistics of stimuli belonging to a common mode (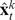). These summary statistics are updated in an incremental, psychologically plausible manner using error-driven learning. Episodes are partitioned into modes by a competitive clustering process similar to mechanisms that have been proposed in many other psychological and neural models [23], [24], [34], [37], [38].

### Model behavior

Eq. 6 operationalizes the idea that large prediction errors will lead to the inference of a new mode: For an old mode the Gaussian log-likelihood is inversely proportional to 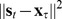, the distance between the current observation and the state when the mode was last active, where 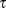 is the time at which the old mode last occurred, while for a new mode the log-likelihood is proportional to 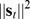 (with the constant of proportionality scaling these distances by the variances of the modes). Thus when 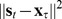 is large relative to 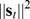 the DP-KF will tend to assign observation *t* to a new mode, analogous to the process by which Blumenfeld et al. 's [22] saliency-weighted learning rule creates a new attractor when the input pattern fails to match any of the existing attractors. (Although the likelihood for a new mode depends on the absolute scale of 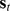, in our simulations this dependence was very weak, as the variance parameter was set to *c* = 1000.) Furthermore, because the variance of a mode grows with the length of time since its last occurrence (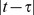), older modes will be more “tolerant” of prediction errors.

**Figure 1:**
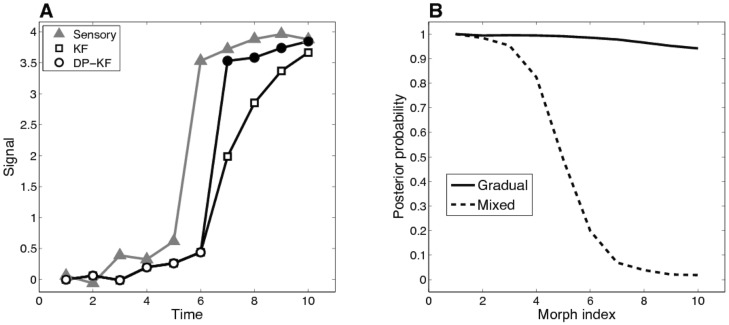
Simulations. (*A*) Simulated sensory measurements and inferred state variables. For the DP-KF, the colors indicate the mode assignment with the highest posterior probability, white circles  =  mode 1, black circles  =  mode 2. (*B*) Posterior probability of mode 1 as a function of morph index in the gradual and mixed protocols, using the DP-KF (averaged over multiple simulation runs). See text for details.

Figure 1A illustrates the results of inference using our model with a one-dimensional sensory stimulus. Here we assumed 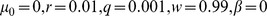 and *α* = 1. The sensory stimulus changed gradually, then underwent a jump, and then changed gradually again. On each time point we first inferred the hidden state based on past observations only (these are the model predictions). Following that, the sensory measurement was observed, thereby allowing the computation of its likelihood and updating of the posterior distribution. As a result, model predictions lag behind the jump. Nevertheless, due to inferring a new mode after the jump, the DP-KF (circles) “catches up” with the sensory evidence after one trial, whereas the regular KF model (squares) takes much longer. This occurs because the KF smooths across the jump as all observations are assumed to be generated by one slowly diffusing mode, whereas the DP-KF achieves piecewise smoothness by segmenting the time series into two modes, thereby producing better predictions.

Figure 1B shows the results of applying the DP-KF to the “gradual” and “mixed” experimental protocols described in the Introduction [15]–[17], [20], [21], [39]. Here we used a sequence of one-dimensional measurements morphing between 0 and 1. In the gradual protocol, the sensory measurement (morphs) increased monotonically with time, whereas in the mixed protocol the morphs were presented in scrambled order. To analyze the simulated data, we re-sorted the indices from the mixed condition to match the gradual condition and calculated the posterior probability of mode 1 for each morph. Consistent with the psychophysical and neurophysiological data [15]–[17], [20], [21], [39], the mixed protocol results in morphs being assigned to two different modes, whereas the gradual protocol results in all the morphs being predominantly assigned to a single mode.

Note that even if each of the modes is already firmly ingrained (through extensive experience with the morphs, as was the case in some of the experimental work we discussed), we still expect to see gradual or abrupt changes in the posterior probability of mode 1 depending on the morph sequence, since the sensory data are ambiguous with respect to the underlying dynamical mode. In other words, the time course of the posterior reflects uncertainty about which mode is currently active, and this uncertainty may change smoothly or abruptly depending on the stimulus sequence.

### Experiment: Memory for dynamically changing visual stimuli

**Figure 2:**
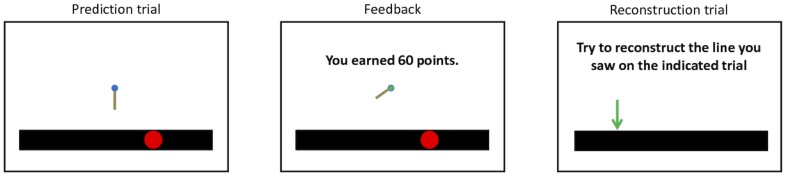
Experimental task. (*Left*) Prediction trial: participants were asked to predict the orientation and length of the next line segment (prediction shown in the center of the screen). At the bottom of the screen, a black circle superimposed on a timeline (the black bar) was used to indicate the trial's serial position in the block. At the start of each block, the black circle started out in the leftmost position; after each trial, the circle's position shifted one position to the right. (*Middle*) After making a prediction, participants were shown the true line segment and received a point score based on their prediction accuracy. (*Right*) Reconstruction trial: at the end of each block, participants were asked to reconstruct from memory the line they saw on one of the first three trials (indicated by an arrow on the timeline). No feedback was given for these reconstruction trials.

**Figure 3:**
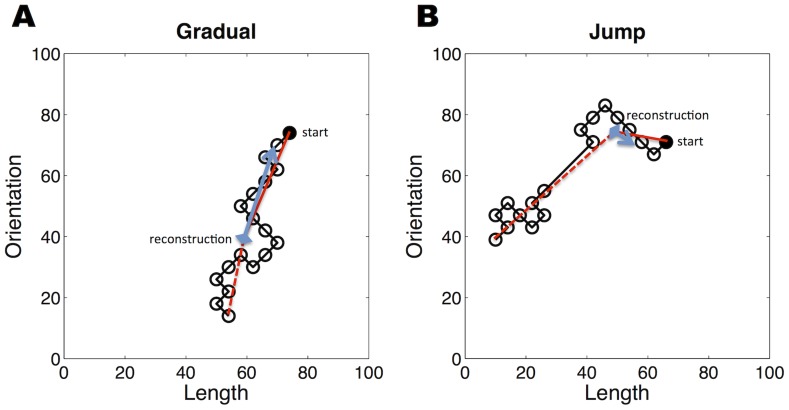
Example trajectories and hypothetical reconstructions. Each circle represents a line segment presented in a sequence, with the shaded circle indicating the first trial. The dimensions are standardized to a [0,100] range. The blue diamond represents a hypothetical reconstruction of the line segment indicated by the arrow. In solid black is the distance between the reconstruction and the starting point, while the dashed black line shows the distance between the reconstruction and the end point. (*A*) A gradual trajectory. Here we expected the reconstruction to be pulled away from the start point and towards the end point. (*B*) A jump trajectory. Here we expected the reconstruction to stay in the vicinity of the pre-jump points. As a result, we expected the distance between the reconstruction and the start point to be smaller in the jump condition as compared to the gradual condition, and the distance between the reconstruction and the end point to be smaller in the gradual condition.

We now describe an experiment designed to test a fundamental prediction of our model: if different modes correspond to different memories, inference of a new mode should protect the memory for old observations from retroactive interference due to new observations (see Materials and Methods for more details). Figure 2 illustrates the task. We exposed human participants to sequences of simple visual stimuli (lines) whose orientation and length changed from trial to trial, and asked them, at the end of the sequence, to reconstruct from memory one of the stimuli from the beginning of the sequence. To ensure that participants were encoding the stimuli, and to provide data that can be compared to the model's trial-by-trial predictions for the purpose of model fitting, we also asked participants to actively predict the orientation and length of the next line. Each participant was exposed to sequences belonging to two conditions: in the “gradual” condition, the lines changed slowly, through small perturbations in orientation/length space; in the “jump” condition, this slow change was interrupted by a large change in the middle of the sequence (Figure 3). Importantly, we kept the overall distance (in terms of orientation and length) between the start and end points of each sequence approximately equal in both conditions.

We reasoned that if participants used prediction errors to segment their observations into distinct modes, then they would infer two modes in the jump condition (one for the first half and one for the second half of the sequence), but only one mode for the gradual condition. Segmenting the sequence would mean that the memory for the first half should be less biased by observations in the second half. We therefore hypothesized that reconstructions of early lines would be more veridical in the jump condition. By contrast, in the gradual condition, later observations would have been assigned to the initial mode, leading to alteration of that mode. Compared to the jump condition, reconstructions in the gradual condition should therefore be more similar to lines observed later in the block, and less similar to the target early lines. Example trajectories and reconstructions for a single participant are shown in Figure S1.

**Figure 4:**
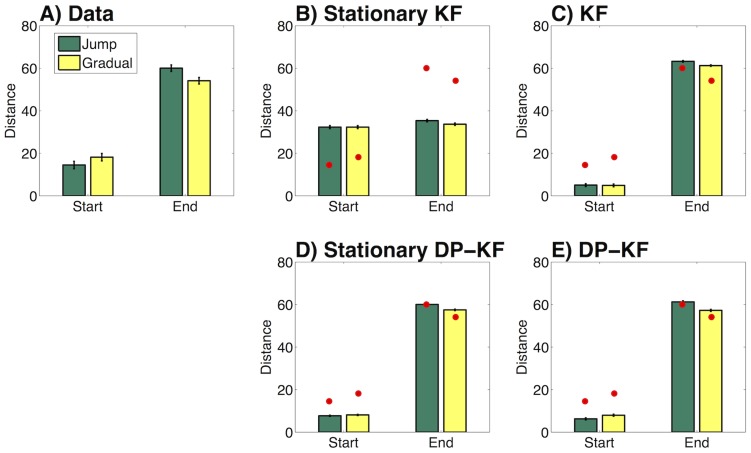
Experimental results and model predictions. (A) Euclidean distance between participants' reconstructions and the observed (true) first and last lines in a block. Error bars represent within-subject standard error of the mean. The results show that participants were more accurate in their reconstructions in the jump condition as compared to the gradual condition. (B) Stationary Kalman filter (KF) model predictions. Data in (A) are represented by black circles. (C) Non-stationary KF model predictions. (D) Stationary Dirichlet process Kalman filter (DP-KF) model predictions. (E) Non-stationary DP-KF predictions.

To test our hypothesis, for each sequence we calculated the Euclidean distance between the participant's reconstruction and the true line observed at the beginning of the block, as well as the distance from the line observed at the end of that block. The results, presented in Figure 4A, show that participants' reconstructions were closer to the last line (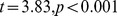), and farther from the first line (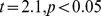) in the gradual condition as compared to the jump condition. A two-way (first/last × gradual/jump) ANOVA confirmed that the interaction was significant (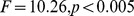). We interpret this result as showing that, in the gradual condition, participants inferred one mode, thereby causing lines from the second half to influence memory for the lines from the first half; by contrast, in the jump condition participants inferred separate pre-jump and post-jump modes, thereby protecting their memory of the pre-jump lines from being distorted by the post-jump lines.

### Model-based analysis of experimental data

Assuming that the individual trace of each stimulus is noisy (see Materials and Methods), it is reasonable for the memory system to use information from multiple trials to aid in reconstruction. In our model, this is accomplished at retrieval by “smoothing” over (or blurring together) the traces of trials that occurred nearby in time. This blurring removes noise under the assumption that stimuli change slowly over time and hence the underlying signal is temporally autocorrelated (whereas the noise is not). Formally, this corresponds to a form of Kalman smoothing [40]. However, it is important to not smooth over instances that are very different from each other (i.e., across time points where an abrupt jump occurred and as a result the signal is no longer autocorrelated). Inference over multiple dynamical modes remedies this problem by segmenting the time series into parts that are each internally smooth; our smoothing algorithm operates within but not across these modes (note that even when there is only a single dynamical mode, smoothing can still reconstruct individual stimuli, rather than blurring them all together, because a representation of each stimulus is available to the retrieval system). A formal description of this smoothing algorithm is given in the Materials and Methods.

 To test how well our proposed model fit participants' data throughout the experiment, we fit several variants of the DP-KF and KF models to participants' responses on prediction trials (in which participants had to predict the next version of the line), holding out the responses on reconstruction trials for validation and comparison between the models (see Materials and Methods for details of the model-fitting methods). Four model variants were constructed from the full model by restricting parameter values as follows:

- **Stationary KF**: A Kalman filter in which the hidden state is stationary (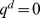 for all *d*). This means all variation is attributed to the sensory and response noise. This model has five free parameters: 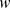, 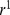, 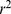, 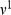, and 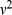 (where superscripts 1 and 2 refer to the two stimulus dimensions: length and angle). The 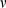 parameters represent response noise variances (see Materials and Methods for more details).
- **KF**: A Kalman filter in which the hidden state is allowed to diffuse over time (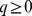). This model has seven free parameters: 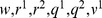, and 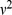.
- **Stationary DP-KF**: In this model, the hidden state can be drawn from multiple modes, where each mode's hidden state is stationary in time (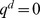 for all *d*). Modes tend to persist over time with the strength of persistence determined by 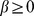. This model thus has seven free parameters: 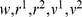, 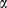 and *β*.
- **DP-KF**: This is the full Dirichlet process Kalman filter model. It allows multiple diffusing modes that each can change over time (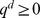). This model has nine free parameters: 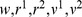, 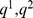, *α* and *β*.

Figure 4B-E shows the predicted reconstruction biases for each of these models. Unlike our participants, neither the KF models nor the stationary DP-KF model showed a cross-over interaction between jump/gradual and start/end. In contrast, the DP-KF model showed a cross-over interaction effect (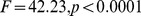). Thus among the four alternatives, only the DP-KF model adequately captured the experimental results.

**Figure 5:**
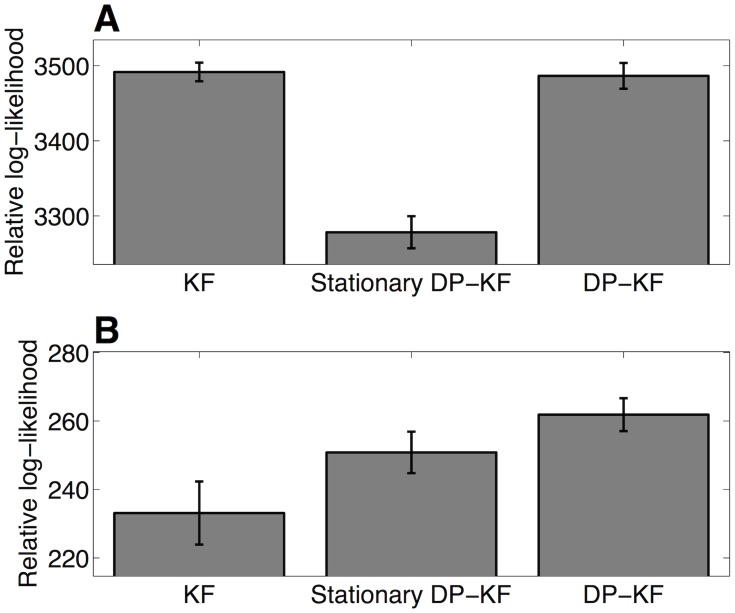
Model comparison. (*A*) Predictive log-likelihood for each model, on prediction trials, relative to the stationary KF model. Larger values indicate superior performance on held-out data from prediction trials. (*B*) Predictive log-likelihood for each model, on the reconstruction data, relative to the stationary KF model. Error bars represent within-subject standard error of the mean.

We quantitatively compared the fits of the different models in two ways. First, we performed cross-validation by splitting the blocks into two halves (even- and odd-numbered blocks), fitting the model to the trial-by-trial prediction data for one half of the blocks and computing the predictive log-likelihood of data for the other half of the blocks. Figure 5A shows the predictive log-likelihood of each model relative to the stationary KF model. The KF and DP-KF models performed similarly (a paired-sample *t*-test revealed no significant difference, 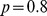), and significantly better than their stationary variants (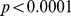).

**Figure 6:**
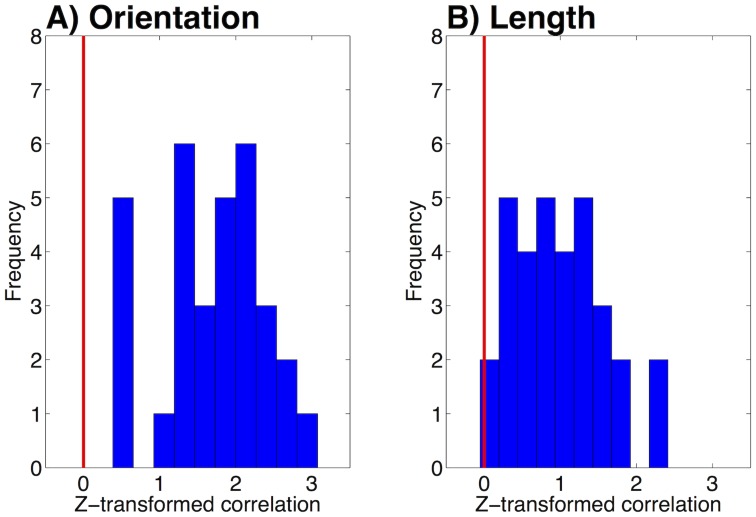
Comparison of model and human reconstructions. Histogram of z-transformed correlations between human reconstructions and model reconstructions for (A) the orientation dimension and (B) the length dimension. Vertical black line indicates a correlation of 0.

Our second model-comparison metric was the predictive log-likelihood of participants' reconstructions. Note that the models were not fit to the reconstruction data, so there is no need to penalize for model complexity: overfitting the prediction-trials data due to too many degrees of freedom will automatically lead to poorer results when trying to predict the reconstruction trials. Figure 5B shows the predictive log-likelihood of each model relative to the stationary KF. According to this measure, the DP-KF model outperformed both the KF variants (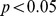) and performed marginally better than the stationary DP-KF (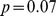). To illustrate the DP-KF model's accuracy in predicting reconstructions, we computed the Pearson correlation coefficient between the human and model reconstructions for each participant separately, Fisher z-transformed this value, and performed a *t*-test against 0 for all participants. Correlations for both orientation and length were significant (each 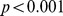, two-tailed *t*-test; Figure 6).

**Figure 7:**
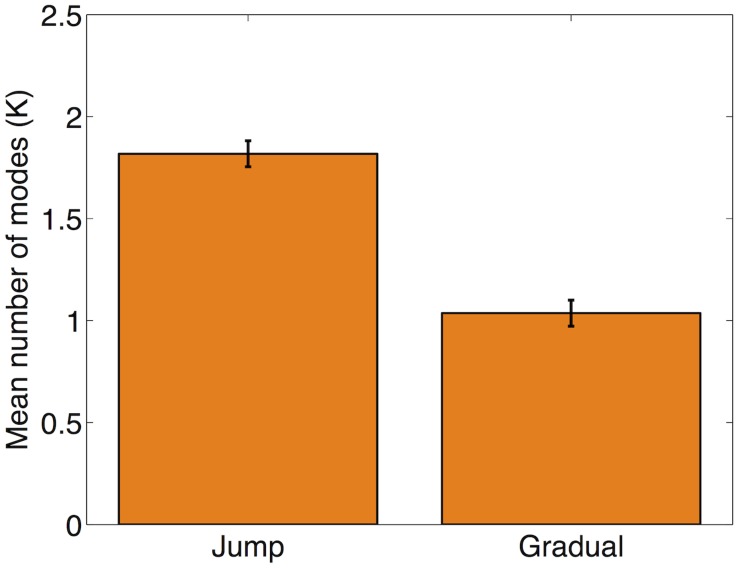
Model-based analysis. Number of modes (K) inferred by the DP-KF model for each condition.

Finally, in keeping with our theoretical predictions, we found that the number of modes (*K*) inferred by the fitted DP-KF model was, on average, higher in the jump condition than in the gradual condition (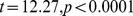; Figure 7).

## Discussion

We addressed, both theoretically and experimentally, a basic question about memory: When does new experience cause an existing memory to be modified versus a new memory to be formed? Our answer took the form of a rational analysis [10]. In particular, we proposed that the structure of memories reflects a process of optimal filtering in a dynamically changing environment, where each memory encodes a distinct “dynamical mode” of the environment. New modes are inferred when there are abrupt discontinuities in the temporal dynamics of sensory data that cannot be explained by existing memories. Such discontinuities are typically accompanied by a large prediction error, suggesting a biologically plausible mechanism for implementing memory-trace formation: The brain may split off new memory traces when large prediction errors are registered [23]–[25], [41]. Prediction errors are believed to be computed in many areas of the brain, including area CA1 of the hippocampus [42] and midbrain dopaminergic nuclei [43]. Indeed, predictive coding theories propose that prediction errors are computed throughout the neocortex [44].

Importantly, the specific model used here belongs to a large family of statistical models that instantiate the idea that abrupt, inexplicable changes in the environment result in inference of a new mode [28]. The main contribution of this paper is to provide an experimental test of this principle in the domain of human reconstructive memory. In our experiment, participants were asked to reconstruct from memory a previously encountered visual stimulus, under conditions where the stimulus had since changed over time either gradually or abruptly. We envision inference over dynamic modes of the environment as giving rise to temporally extended episodic memory traces that group together individual stimulus traces, thus causing some generalization or interference between the memories of different specific observations. We thus measured the degree to which later stimuli modify the memory of earlier instances by assessing the extent to which the reconstructed stimulus shifted from the starting point of the stimulus trajectory towards the end point. We showed that gradual change resulted in greater memory modification than abrupt change, in agreement with our theoretical prediction that gradual change would favor inference of a single dynamical mode that would incorporate all stimuli in a block, whereas abrupt change would favor the inference of multiple modes, each relatively untainted by experience that is associated with the other mode.

The behavioral effect that we showed cannot be explained by recency or primacy biases: A recency bias does not predict a difference between the conditions, because the conditions were matched for total distance traveled and for trial-to-trial differences in the stimuli in all trials but the jump trial (which was always in the middle of the sequence). Therefore, stimuli at the end of a block, just prior to the reconstruction trial, were (on average) equally similar to the initial stimulus across conditions. Likewise, a primacy bias does not predict a difference between conditions, since the stimuli in the beginning of the block did not differ systematically between conditions.

The choice between modifying an existing memory versus creating a new one is formalized in our model using a nonparametric prior over partitions known as the Chinese restaurant process [33] (see [31] for an explanation of the Chinese restaurant metaphor and its origins). This prior has previously been used to model category formation [34], [35], Pavlovian conditioning in multiple contexts [24], [45], word segmentation [46] and task-set learning [47] (for a review of this literature, see [48]). All of these domains have in common the problem of segmenting stimuli and actions into coherent clusters (or, in our case, modes). The Chinese restaurant process is a natural prior for segmentation because it allows an unbounded number of clusters while preferring fewer clusters. This prior thus expresses a bias towards simplicity [32]. Even without such a prior bias, simpler segmentations are naturally favored by Bayesian inference due to the “automatic Occam's razor” phenomenon [49], whereby simpler explanations of data have higher marginal likelihood than more complex explanations. While the experiment we report does not directly address whether humans exhibit a simplicity bias in memory formation, this question has been addressed by other work from our laboratory [50].

One limitation of the current study is that it did not test a further prediction of our model: When a change occurs, an old mode can be reinvoked, rather than creating a new mode. Thus our findings could potentially be explained by a model that creates a new mode every time a large change is observed (although previous modes would still have to be maintained in memory to allow recall, unlike some models, e.g., [11]). In future work, we will test the hypothesis that old modes can be modified in this paradigm. Using a perceptual estimation paradigm [50] we have shown that participants can update two modes in an alternating fashion, if these are signaled externally (in that case, by the color of the stimuli). However, unlike our current study, this earlier study did not manipulate the dynamics of stimulus trajectories and so could not address the dynamics of memory formation as a result of (abrupt vs. gradual) change in the environment.

### Related work

Several authors have proposed neural implementations of the KF [51], [52]. Wilson and Finkel [52] derived an approximation of the KF that can be computed by a recurrent neural network when the prediction error is small. Intriguingly, when the prediction error is large, their approximation ‘breaks down’ by creating two bumps in the posterior distribution (rather than one as in the exact KF) with each bump implementing an independent KF. Our theory suggests a normative account of this feature, since a network that creates multiple bumps is precisely what is required by the DP-KF algorithm. Pursuing this connection is an exciting direction for future research.

Work on change detection [11], [53]–[59] addresses a similar question: how does the brain detect a change in the statistics of sensory signals? The study of Nassar et al. [56], for example, showed that humans use the recent history of prediction errors to determine when a change has occurred. This work differs from our own in several ways. First, most existing change-detection theories assume stationary sensory statistics between jumps, whereas we allow for gradual change between jumps. Second, once a jump has occurred, theories of change detection assume that the statistics of earlier epochs are no longer relevant and can be discarded; in contrast, our model assumes that participants are able to retrieve statistics from earlier modes, and in general allows for the environment to return to earlier modes (as noted above, our current experiment did not test this latter property of the model).

Our work also intersects with research in cognitive psychology on the reuse of existing memory traces. For example, repeating items on a list tends to aid their recognition without degrading recognition of other items (the *null list-strength effect* [60]). To explain this, Shiffrin et al. [8] assumed that repetition of items results in refinement of existing traces, rather than formation of new traces. Thus, there must be *some* reuse of memory traces. The question, then, is what counts as a repetition. Visually similar stimuli such as those used in our experiment may be judged by the memory system to be essentially the same item (i.e., a “repetition”). Our theory further asserts that small changes in these “repetitions” drive modification of existing memories, but not formation of new memories. This is similar to what Bower and Winzenz [7] dubbed the “reallocation hypothesis,” according to which inputs are matched to memory traces and incorporated into an existing trace if the match is sufficiently high; otherwise, the input is routed to a new trace (see also [9]). Interestingly, evidence suggests that failure to recognize a new context can sometimes lead to neither outcome: using an auditory statistical learning paradigm, Gebhart et al. [61] found that changes in structural information can go undetected without the aid of additional cues (e.g., sounds marking the transition between structures), preventing participants from learning new structures. This suggests that future models should incorporate a mechanism that allows some information to evade both old and new memories.

The dynamically updated posterior posited by our model bears some resemblance to the drifting context vector posited by several models in the memory literature [62], [63]. For example, the Temporal Context Model (TCM) introduced by Howard and Kahana [63] assumes that list items are bound to a context vector that is essentially an average of recently experienced items. In earlier work [64], we operationalized the context vector as a posterior over latent “topics” that play the same role as modes in the present paper. In our current theory, items are bound to modes in much the same way that items are bound to the context vector in TCM. The connection to TCM also highlights the way in which episodic and semantic memory are deeply intertwined in contemporary theories: “episodic” traces of individual items become bound to “semantic” representations that average over multiple items [65]. Likewise in our model, episodic and semantic components are intertwined: a separate trace for each sensory stimulus is stored, but the traces are effectively blurred together by the smoothing operation during retrieval. Although the idea of separate episodic and semantic memory systems has been very influential [13], it has been known since Bartlett's investigations [66] that semantic knowledge exerts strong constraints on many aspects of episodic memory [67], [68]. A similar rapprochement has emerged in theories of category learning, where “episodic” (exemplar) and “semantic” (prototype) representations are combined to form varying levels of abstraction [35], [69], [70].

Another related line of work concerns the effects of novelty on memory. Our model predicts that a novel stimulus is more likely to be encoded in a separate trace compared to a familiar stimulus, making it less likely that the novel stimulus will suffer interference from other stimuli at retrieval. This prediction has been confirmed many times in the form of the von Restorff effect [71]. Note that while the von Restorff effect reflects proactive interference (older memories interfering with the retrieval of newer memories) and our experiment tested retroactive interference (newer memories interfering with the retrieval of older memories), according to our model these are essentially due to the same process of grouping of different observations into temporally extended episodic memory traces.

The idea of comparing gradual and abrupt changes as a means of influencing memory updating has also been explored in the motor control literature [72]–[74]. For example, Kagerer et al. [72] had participants make arm movements to a target and then introduced a perturbation (by rotating the visual feedback) either gradually or abruptly. Participants adapted to the perturbation; following the removal of the perturbation, participants exhibited an after-effect in which movement errors were in the direction opposite to the perturbation. Kagerer et al. found that the after-effect was smaller for participants in the abrupt condition than in the gradual condition. This pattern of results is consistent with the idea that two separate motor memories were formed in the abrupt condition, thereby allowing the pre-perturbation memory to be reinstated quickly. The larger after-effect in the gradual condition suggests that in that case the gradual perturbation led to modification of the original memory. Such modifications can be long-lasting: Yamamoto et al. [75] have shown that learning a gradually changing motor task produces a motor memory that can be recovered over a year later.

Finally, we have recently reported related findings in the domain of Pavlovian fear conditioning [41]. Rats learned to associate a tone with a foot-shock. Subsequently, one group of rats were presented with the tone in the absence of shock (standard ‘extinction’ of the tone-shock association). A second group of rats experienced the same number of tones, with the the tone-shock contingency only gradually reduced to zero (that is, to full extinction). Although all rats showed similarly diminished fear of the tone at the end of the ‘extinction’ phase, rats in the standard extinction condition exhibited subsequent recovery of fear (as is typically seen after extinction training), whereas rats in the gradual condition showed no evidence of fear recovery. These findings are consistent with the idea that the fear memory is more likely to be modified by extinction training in the gradual condition, thereby reducing the probability of later recovery.

## Conclusions

In this paper, we empirically investigated a fundamental prediction that models of change detection make for memory. If, as we hypothesize, new experience is incorporated into old memories based on similarity, then abrupt change (i.e., dissimilar data) should prompt the creation of a new memory trace, and thus protect old memories from being modified by new data, whereas gradual change will not. Our experimental results confirm this prediction, thereby providing support for a statistical account of how continuous experience is parsed into discrete memory traces. We conclude that memories are not simply a record of our ongoing experiences; the organization of memory traces reflects our subjective inferences about the structure of the world that surrounds us.

## Materials and Methods

### Ethics statement

The experiment was approved by the Institutional Review Board at Princeton University.

### Participants

32 undergraduate students received course credit or payment ($12 per hour) for participating in the experiment. The experiment was approved by the Institutional Review Board at Princeton University.

### Stimuli

The stimuli consisted of oriented line segments that changed in orientation and length on every trial. Each line segment was generated from the previous one by (randomly) adding or subtracting a fixed length (0.89 mm) and a fixed angle (14.4°), thus generating a 45° ‘move’ in an orientation/length space in which one unit was 14.4° and 0.89 mm, respectively. ‘Moves’ were restricted so that the new line segment did not overlap with the previous line segment (that is, there was no ‘backtracking’ in orientation/length space; see Figure 3). Jumps were also at a 45° angle, but traversed a distance 4 times as long as the other steps (i.e., 3.6 mm length and 57.6° angle). Jumps always occurred (if they did) in the middle of the trajectory (between trials 9 and 10), and were unsignaled to the participant. Finally, in generating trajectories through orientation/length space, we required the Euclidean distance between the start and end points to lie within a narrow range (60–70% of the maximum possible distance) regardless of the condition (jump or gradual). Examples of jump and gradual trajectories are shown in Figure 3.

### Procedure

Participants played 12 blocks of the task (6 jump trajectories and 6 gradual trajectories, randomly interleaved). Each block consisted of a sequence of 18 prediction trials. A timeline showed participants the serial position of each trial in a block. On each prediction trial, participants used a mouse to adjust the orientation and length of a line on the screen so as to predict the next observed line. After making their prediction, participants were shown the true line and awarded points based on how accurate their prediction was. The prediction task was aimed at encouraging encoding of the different line segments in memory, and also provided data for fitting our models (see below). At the end of the block, participants were given a reconstruction trial; on this trial, they were shown an arrow pointing toward a point on the timeline and asked to reconstruct the line segment they saw on that trial. Participants were always asked to reconstruct one of the first 3 trials in the block. No feedback was given on reconstruction trials.

### Reconstruction by smoothing

Let 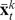 denote the estimated stimulus for time *t* given all observations up to the time of retrieval conditional on 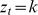. Kalman smoothing [40] constructs this estimate through a backward recursion:
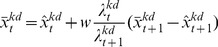
(7)


for each dimension *d*. In essence, smoothing combines the filtered estimate 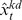 with information from the future propagated backward in time. We take 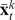 to be the model's prediction for a participant's reconstruction of the stimulus shown at time *t*.

### Model-fitting

Prior to model-fitting, the stimulus values (length and orientation) were rescaled to [0, 100]. To model responses, we assumed that participants report the posterior mean, corrupted by anisotropic Gaussian noise (with variances 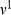 and 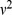, for length and orientation, respectively. Depending on the model variant, the noise variance *r*, the response noise variance *v*, the diffusion noise variance *q*, the stickiness parameter *β* and the concentration parameter *α* were treated as free parameters and fit to each participant's data by minimizing the negative log-likelihood of each participant's predictions using a numerical optimizer (the routine fmincon in Matlab), while constraining parameters to lie in the appropriate range. To prevent implausibly large values of *v*, *q* and *r*, we constrained these to be less than 10, 30 and 20, respectively, although our results do not depend on these precise values. To avoid local minima, the optimization was run from 3 randomly chosen starting points. We assumed that responses were generated from the filtered state estimate (or smoothed state estimate, in the case of retrieval), corrupted by Gaussian noise with anisotropic noise variance (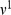 and 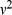). For the KF model, *α* was set to 0. We set the prior covariances to be 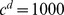, instantiating an approximately uniform distribution over mode starting points. Reconstruction trials were not used in any of the fitting procedures. To model noise in the reconstruction process, we added a constant of 5 to the sensory noise variance (*r^d^*). This value was chosen by hand, but the results were not sensitive to its precise value.

## Supporting Information

Figure S1 **Example trajectories and reconstructions for a single participant.** The top row shows trajectories in the “gradual” condition. The bottom row shows trajectories in the “jump” condition. The first trial is indicated by the large circle, and the blue diamond shows the reconstruction.
(EPS)
